# Unusual kinematics of the Papatea fault (2016 Kaikōura earthquake) suggest anelastic rupture

**DOI:** 10.1126/sciadv.aax5703

**Published:** 2019-10-02

**Authors:** A. Diederichs, E. K. Nissen, L. J. Lajoie, R. M. Langridge, S. R. Malireddi, K. J. Clark, I. J. Hamling, A. Tagliasacchi

**Affiliations:** 1 School of Earth and Ocean Sciences, University of Victoria, Victoria, BC, Canada.; 2 Department of Geophysics, Colorado School of Mines, 1500 Illinois St., Golden, CO, USA.; 3 GNS Science, PO Box 30 368, Lower Hutt 5040, New Zealand.; 4 Department of Computer Sciences, University of Victoria, Victoria, BC, Canada.

## Abstract

A key paradigm in seismology is that earthquakes release elastic strain energy accumulated during an interseismic period on approximately planar faults. Earthquake slip models may be further informed by empirical relations such as slip to length. Here, we use differential lidar to demonstrate that the Papatea fault—a key element within the 2016 M_w_ 7.8 Kaikōura earthquake rupture—has a distinctly nonplanar geometry, far exceeded typical coseismic slip-to-length ratios, and defied Andersonian mechanics by slipping vertically at steep angles. Additionally, its surface deformation is poorly reproduced by elastic dislocation models, suggesting the Papatea fault did not release stored strain energy as typically assumed, perhaps explaining its seismic quiescence in back-projections. Instead, it slipped in response to neighboring fault movements, creating a localized space problem, accounting for its anelastic deformation field. Thus, modeling complex, multiple-fault earthquakes as slip on planar faults embedded in an elastic medium may not always be appropriate.

## Teaser

Differential lidar reveals unusual fault characteristics within a complex earthquake.

## INTRODUCTION

**Fig. 1:**
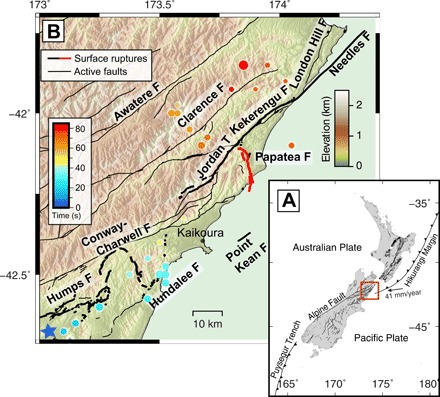
Map of the 2016 Kaikōura earthquake and surrounding area. (**A**) Transpressional tectonic setting of the northeastern South Island of New Zealand. (**B**) Map of surface ruptures from the 2016 M_w_ 7.8 Kaikōura earthquake, shown in bold black lines with the Papatea fault in red (*8*, *28*). Dots represent scaled relative energy release from back-projection results (*15*) and are colored by time since rupture onset. Mapped active faults that did not rupture during the Kaikōura event are indicated by thin black lines (*28*).

Complex multiple-fault earthquakes offer vital insights into the physics of rupture propagation and arrest, which are important for seismic hazard analysis and rupture forecasting (*1*–*5*). However, characterizing such events can be challenging due to complex seismic and geodetic signals that result in parameter trade-offs between neighboring fault segments in rupture models. These problems are well illustrated by the 2016 M_w_ 7.8 Kaikōura earthquake, which cascaded across a network of dextral, sinistral, oblique, and reverse faults in the northeastern South Island, New Zealand (Fig. 1). Published source models based on a combination of seismic, satellite geodetic, and field data exhibit a wide variety of fault geometries and complexities, with as few as 2 (*6*, *7*) to over 20 (*8*) discrete rupture segments. Consequently, there are disagreements over fundamental characteristics of the earthquake, such as the dimensions between rupture segments separating the major crustal faults, and the degree to which the underlying Hikurangi subduction interface slipped coseismically.

**Fig. 2:**
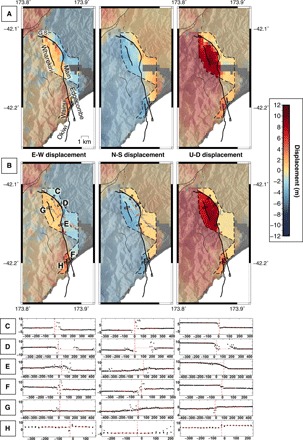
3D displacement fields around the Papatea fault with sample fault-perpendicular profiles. (Left) E-W, (middle) N-S, and (right) up-down surface displacement fields around the Papatea fault from (**A**) SAR measurements [~330 m × 450 m pixel resolution, from Hamling *et al.* (*4*)] and (**B**) D-lidar calculations (25 m) within the dashed line polygon. Positive displacement directions are east, north, and up. Coarser displacement pixels outside the polygon of double lidar coverage are SAR measurements (*4*). G.S. labels the location of the George Stream. Thick black lines are Papatea surface ruptures. (**C** to **H**) Example 100 m × 900 m fault-perpendicular profiles [black rectangles in (B)] through the *x* (left), *y* (middle), and *z* (right) lidar surface displacement fields. The vertical axis is displacement, and the horizontal axis is distance, both in units of meters. Each black dot is a single-cell displacement, vertical dashed red lines show the fault scarp, and horizontal red lines show extrapolated linear fits of chosen data points with 50% confidence bounds (dashed).

The kinematics of the 19-km-long, south-striking Papatea fault and its role in the Kaikōura rupture are especially enigmatic (Fig. 1B). Field surveys documented reverse-sinistral offsets of up to ~10 m on the Papatea fault, far exceeding historical slip-to-length ratios of large earthquakes (*9*). Coarse three-dimensional (3D) surface displacements resolved from interferometric synthetic aperture radar (InSAR) and synthetic aperture radar (SAR) pixel offsets (Fig. 2A) indicate multimeter uplift and counterclockwise vertical-axis rotation of a ~15 km × 100 km “Papatea block” in the hanging wall of the Papatea fault (Fig. 6). Relative motion of this block, with respect to neighboring areas, was poorly fit by initial elastic models (*4*). Slip on the Papatea fault is poorly resolved seismically [e.g., (*1*, *7*)] and is associated with a notable scarcity of aftershocks (*10*), leaving its subsurface geometry largely unknown. Consequently, published models of the Kaikōura earthquake implement a wide variety of assumed geometries or simply exclude it altogether.

To investigate further, we used a rarely available pair of pre- and postearthquake airborne lidar surveys and a new implementation of the iterative closest point (ICP) algorithm (*11*–*13*) to map the 3D surface displacement field along the onshore Papatea fault (see Materials and Methods). By profiling this displacement field (*14*), we resolve the slip vector, fault dip, and rake at discrete locations along four Papatea fault ruptures. In doing so, we reveal the Papatea fault as a twisting, mostly high-angle reverse-sinistral fault, where fault dip and dip direction vary along strike. The resulting surface deformation field cannot be reproduced using conventional elastic models (*4*). We conclude this fault rupture occurred in rapid response to shortening caused by neighboring elastic ruptures, rather than due to the release of accumulated interseismic strain along the Papatea fault itself, as is conventionally assumed. This may explain why the fault appears seismically quiescent in many back-projection models [e.g., (*7*, *15*, *16*)]. These unusual patterns in fault kinematics and anelastic surface deformation call into question assumptions about fault elasticity in complex multifault earthquakes.

### The 2016 Kaikōura earthquake

The Kaikōura earthquake occurred on 14 November 2016 at 12:03 a.m. local time (11:03 on 13 November UT) in the North Canterbury domain and Marlborough Fault System of the South Island of New Zealand (Fig. 1A) (*8*, *17*). This region of transpressional tectonics marks the transition between the Hikurangi margin, where Pacific oceanic lithosphere subducts westward at ~48 mm/year beneath the Australian plate, and the Alpine Fault and Southern Alps, an oblique collision zone between continental portions of the same plates [e.g., (*18*, *19*)]. The North Canterbury domain is characterized by relatively slow-slipping reverse faults, whereas the Marlborough Fault System contains an anastomosing array of faster-slipping (up to ~25 mm/year) dextral strike-slip and thrust faults. This earthquake was the largest ever recorded in this region, with the previous strongest (the 1848 Blenheim earthquake) estimated at M_w_ 7.5 (*20*).

The 2016 earthquake ruptured at least 20 faults, 13 of them with multimeter slip, making this among the most complex earthquakes ever recorded globally (*4*, *8*). It nucleated at ~15-km depth on the Humps fault, as an oblique thrust fault in the North Canterbury domain, before rupturing eastward onto the neighboring Leader and Hundalee faults (Fig. 1B). Subsequent rupture of an offshore fault or faults may be responsible for a local tsunami with up to ~5 m run-up (*21*, *22*). It then propagated or jumped northward onto the Marlborough Fault System, where most of the seismic moment was released and where the largest surface slip was documented on the Jordan, Papatea, and Kekerengu faults (*4*, *8*, *23*, *24*). The earthquake terminated after ~80 to 100 s on the Needles fault within the Cook Strait, ~180 km northeast of the epicenter [e.g., (*7*, *15*, *22*, *23*, *25*–*27*)].

The arcuate, ~19-km-long Papatea fault (Figs. 1B and 2A) strikes ~south-southeast overall between the coast and George Stream, where it converges with the ~SW-NE–striking Jordan and Kekerengu faults, and offshore Waipapa Bay, where it may converge with other offshore faults (*8*, *28*). The longer onshore section approximately follows the trace of the lower Clarence River, with steep hills in its western hanging wall and more subdued terrain in its eastern footwall. Although mapped geologically, the Papatea fault was not considered to be active prior to the 2016 Kaikōura earthquake (*29*, *30*).

Langridge *et al.* (*28*) mapped 2016 surface ruptures along ~16 km of the main Papatea fault strand, a short coastal fault east of the main strand, called the Edgecombe trace, and a discontinuous western strand made up (from north to south) of the Wharekiri, Back-basin, Wainui, and offshore Okiwi ruptures (Fig. 4). They found maximum and average throw across the main strand to be 9.5 ± 0.5 and 4.5 ± 0.5 m, respectively, with maximum sinistral offsets of 6.1 ± 0.5 m. At the coast, Clark *et al.* (*21*) measured ~4-m vertical offsets on the Wainui trace and main strand from differencing lidar digital elevation models (DEMs), with the narrow block in between uplifted by ~6.6 m from sea level. From satellite optical imaging of horizontal deformation across the Papatea fault damage zone, Klinger *et al.* (*31*) observed strain asymmetry consistent with northward rupture directivity, suggesting that the fault provided a linkage between southern offshore faults and the northern Jordan and Kekerengu faults (*31*, *32*). One field measurement of the fault dip has been collected along the main strand at a clear cross-sectional exposure across the Clarence River, indicating a dip of 51°W (*28*). Coastal field measurements along the Wainui trace indicate a near vertically dipping western strand (*28*). 3D surface displacements from satellite photogrammetry now cover the entirety of the 2016 surface rupture, but a detailed analysis of these results has been focused on other faults (*24*).

With such poor constraints on its subsurface dip, most early attempts at modeling the Kaikōura earthquake excluded the Papatea fault altogether (*4*, *6*, *7*, *21*, *22*, *25*, *26*). Subsequent modeling studies have implemented the Papatea fault in a wide range of configurations—from listric structures that grade into shallow (17°) angles at depth (*32*) to moderately dipping (47° to 70°) planar geometries (*15*, *16*, *23*, *24*, *27*, *29*–*31*, *33*, *34*). In addition to this, many studies do not clearly explain how modeled fault dips were chosen or whether they were fixed during inversion. If the Papatea fault is misrepresented in Kaikōura earthquake models, then its seismic and surface deformation signals may be misinterpreted as sourced from other nearby faults. This is emphasized by the fact that some models place maximum subduction interface slip beneath the Papatea block [e.g., (*22*, *23*)].

## RESULTS

**Fig. 3:**
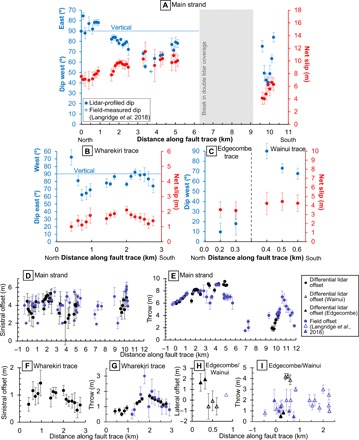
Fault kinematics from rupture profiling. Lidar-derived net slip (red) and fault dip (blue) measurements for the (**A**) main strand, (**B**) Wharekiri trace, and (**C**) Edgecombe and Wainui traces of the Papatea rupture. Uncertainties in slip (pink bars) are calculated from 50% limits in displacement drawn from each swath profile. Uncertainties in fault dip (light blue bars) are ±1 σ values of a distribution of dips yielded from a Monte Carlo simulation. Lateral and vertical slip components along strike of the Papatea fault strands (black datapoints, with ±2 σ uncertainties) and from field and lidar elevation change measurements (*28*) (purple datapoints with uncertainties where available) for the (**D** and **E**) main strand, (**F** and **G**) Wharekiri trace, and (**H** and **I**) Edgecombe (sinistral, solid triangles) and Wainui (dextral, open triangles) traces. Distances along the horizontal axes are from north to south of each fault trace and correspond to the 1-km markers (gray dots) in Fig. 4.

We used pre- and postearthquake airborne lidar to map 3D surface displacements along the Papatea fault and profiled the deformation field (Fig. 2) to map slip vector and dip distributions (*11*–*14*) (Materials and Methods). Figure 3 shows net slip (red) and fault dip (blue), as well as the lateral and vertical components of the slip (black) as a function of distance along the strike, for all profiled fault traces. All results are tabulated in table S1.

### Fault geometry of the main strand

**Fig. 4:**
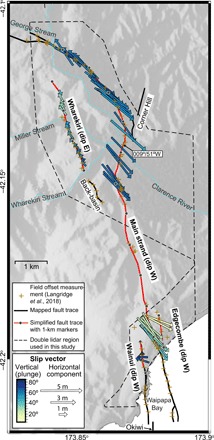
Map of the Papatea fault zone. Lidar-derived fault slip vectors (arrows) colored by plunge, showing hanging wall motion relative to footwall (note vectors flip with changing dip direction). Slip vectors were measured from swath profiles taken perpendicular to the simplified fault traces in red, which closely approximate the published, mapped fault traces shown in black (*28*). Gray dots along the red line mark 1-km distance increments along each fault strand (starting from 0 km at each northern end) and correspond to the horizontal axes in Fig. 3. The black dashed line polygon shows the region of double lidar coverage considered in this study. Field measurement localities (*28*) are indicated by brown plus signs.

We collected 34 slip, dip, and rake measurements along the main strand of the Papatea fault, using a simplified trace (Fig. 4) to approximate the fault. The main strand is characterized by multimeter vertical (west side up; Fig. 3E) and left-lateral offsets (Fig. 3D), but we found that the fault geometry changes substantially along the strike. At its northern end (~0 to 1 km distances on Fig. 3A), it accommodates a net slip of ~7 to 8 m, with vertical and left-lateral components of ~6 to 7 and ~2.5 to 4.5 m, respectively. This section of the fault is subvertical, as indicated by large vertical displacements with horizontal slip vectors that closely parallel the fault (Fig. 4). Moving southward, slip increases on a progressively shallowing, W-dipping fault plane, culminating at ~3.5 km along the strike with peak slip of ~11.5 m, coeval with a local minimum in fault dip of ~56°W. This measurement point is close to the exposure across the Clarence River, with a measured dip of ~51°W (*28*). Further south, the fault steepens to ~70° to 80° at ~5-km distance while retaining a high slip of 9 to 11 m and attaining a peak throw of ~9.5 m. South of ~6 km along the strike, lidar coherence or coverage is lost for a distance of ~4 km. Accounting for changes in dip direction at its northern tip, the northern ~5 km of the main strand has average and SD values in net slip of 9.0 ± 1.3 m, throw 7.8 ± 1.1 m, left-lateral slip 3.9 ± 0.9 m, dip 81°W ± 12°, and rake 64° ± 6°. The rather narrow parameter ranges are despite a pronounced curvature of up to 73° in fault strike.

Near the middle of the main strand fault trace, ~6 km from its northern end, there is a ~4-km gap in double lidar coverage. South of this gap, the double lidar coverage along the coast captures a ~1-km-long fault section (see dashed polygon in Fig. 4). Here, we obtained very similar horizontal slip vectors to the northern fault section, but much smaller vertical offsets (averaging 2.8 ± 1.0 m) and consequently shallower dip values (58°W ± 17°). We also determined average and SD values in net slip of 5.4 ± 1.1 m, left-lateral slip of 4.1 ± 0.6 m, and rake of 40° ± 7°. Along-strike gradients in slip, dip, and rake greatly exceed those of the northern main strand section, likely reflecting that the coastal section is part of a step-over zone in which slip is transferred westward onto the Wainui and offshore Okiwi traces.

### Fault geometries of the Wharekiri, Wainui, and Edgecombe traces

We collected 15 slip, dip, and rake measurements along the north-northwest (NNW) striking, eastward-dipping (Fig. 3B) Wharekiri trace in the hanging wall of the main strand. The Wharekiri rupture is characterized by clear vertical (east side up, Fig. 3G) and sinistral offsets (Fig. 3F) over a distance of ~3 km, but offsets in the east-west (E-W) displacement field are much harder to discern (Fig. 2B). Peak net slip of ~2.1 m, throw of ~1.7 m, and left-lateral slip of ~1.2 m are observed ~2 km along the measured section. Slip vectors point consistently toward the NNW, subparallel to the rupture trace (Fig. 4). We calculated average and SD values of 1.5 ± 0.3 m in net slip, 1.2 ± 0.4 m in throw, 0.9 ± 0.3 m in left-lateral slip, 81°E ± 12° in dip, and 52° ± 12° in rake.

We analyzed ~1-km sections of the westward-dipping Wainui and Edgecombe traces, on either side of the coastal section of the main strand. The Wainui trace is most evident in the vertical displacement field (Fig. 2B), with east side up sense; we determined up to ~4.4 m of predominantly normal slip on a steep (68° to 90°) west-dipping fault (Fig. 3, C, H, and I). The Edgecombe trace shows less scatter in the horizontal components; we estimated up to ~3.5-m sinistral-thrust motion on a low-angle westward-dipping fault (Fig. 3, C, H, and I).

## DISCUSSION

### Comparison to field measurements

In general, our offset measurements are in reasonable agreement with those measured in the field or from lidar elevation change maps by Langridge *et al.* (Fig. 3) (*28*). One exception is along the eastward-dipping Wharekiri trace, where we found consistent left-lateral offsets of 0.4 to 1.4 m and maximum throw of ~1.7 m, but Langridge *et al.* (*28*) reported no field evidence for lateral displacement and an isolated peak of ~3.0 m in throw. A second exception is along the Wainui trace, where we observed large (~4 m) throw and small (~0.5 m) dextral motion, but Langridge *et al.* (*28*) reported smaller (<3 m) throw and small (~0.5 m) sinistral motion. Along all faults, our measured offsets vary more smoothly along strike with lower scatter than those of Langridge *et al.* (*28*). This is especially true of the lateral components (Fig. 3). These were measured in the field by extrapolating piercing lines (mostly cultural features such as fences and roads) within ~100 m of the primary rupture trace (*28*), close to or perhaps even within the fault damage zone. In contrast, by using swath profile displacements over an aperture of 900 m and extrapolating only the “far-field” linear data trends, we captured the cumulative offset across the fault damage zone. Simple elastic forward models suggest that our profiles are most sensitive to slip in the upper ~2 km. Nevertheless, our measurements are not consistently larger than those of Langridge *et al.* (*28*), implying that any shallow slip deficit is small. Mackenzie and Elliot (*35*) pointed out that along oblique faults, unrecognized fault-perpendicular slip components (heave) can influence the apparent lateral offset of piercing lines, leading to measurement biases that scale with the obliquity of the piercing line to the fault. Since heave is often the most difficult slip component to survey in the field—particularly when it is reverse sense—we suspect that this bias may have influenced some of Langridge *et al.*’s (*28*) most scattered measurements. In contrast, our own lateral offsets are extracted from the displacement field, rather than from piercing lines, and are not prone to such biases. Langridge *et al.*’s (*28*) vertical offsets are generally smoother and closer to our own, which reflects that they differenced the same paired differential lidar data as the main procedure to determine throw.

### “Twisted” fault geometry

Our measurements of fault dip are of mixed consistency with values reported by Langridge *et al.* (*28*). Our average dip for the Wharekiri trace of 81°E confirms Langridge *et al.*’s (*28*) suspicion that this fault is steeply dipping, and our average dip for the Wainui trace of 77°W is close to their average field measurement of 84°W. On the main strand, Langridge *et al.*(*28*) estimated a 55°W ± 10° fault dip. The closest lidar profile-derived dip measurement is within a few degrees of this single-field data point, at 56°W. However, this point represents a local minimum in dip along strike, and we found a much steeper average dip of 81°W within the northern Clarence Valley section as a whole. At the coast, the main strand alone dips 58°W, but far-field displacements suggest that the three coastal faults converge or merge at depth into a more gently dipping structure, with estimated dip ~25 to 48°W (fig. S2).

Collectively, our results indicate substantial variations in fault dip along strike. If the Wharekiri and Wainui traces belong to the same fault, as Langridge *et al.*(*28*) suggest, then this structure twists from steeply E-dipping in the north to steeply W-dipping at the coast, with consistent relative uplift of the eastern side of the fault. The Papatea fault shows even greater variability, dipping subvertically at the northern end of the profile, ~60°W at ~3 to 4 km, and 70° to 80° at ~5 km along strike. At the coast, the bulk fault zone dips just 25° to 48°W. Thus, the Papatea fault cannot be characterized as a single planar structure, as it is represented in the majority of the published Kaikōura earthquake rupture models. To capture accurately these short-wavelength geometrical changes, we suggest that future modeling studies explore parametrizing the Papatea fault as multiple fault planes that “twist” from subvertical dips in the north to much gentler dips at the coast. Despite these dip variations, as well as coincident changes in fault strike, we note that the horizontal slip vector shows remarkable consistency in azimuth and length along all of the main strand, suggesting coherent southeastward motion (as well as uplift) of the Papatea block relative to the eastern footwall (Fig. 4).

Our results also demonstrate consistencies with those of Zinke *et al.* (*24*), who derived 3D surface deformation fields for the Kaikōura earthquake using optical image correlation and estimated fault geometries of a number of rupture strands, including the Papatea and Wharekiri faults. Their calculated median dip of 65°W ± 20.4°, peak net slip of 11.0 m, and a maximum fault dip of 89.1°W along the main strand are in reasonable agreement with our results. Their average net slip of ~1.8 m along the Wharekiri trace also agrees well with our results, although their average dip of 56° ± 23.6° is shallower. We do note that our own lidar-derived estimates of fault dip demonstrate lower scatter with greater sample density from finer-resolution displacement maps, although, of course, Zinke *et al.*’s (*24*) displacement fields capture the entire rupture zone.

### Unusual kinematics

With peak throw of ~9.5 m and maximum fault dip near vertical, rupture of the ~19-km-long Papatea fault in the 2016 Kaikōura earthquake violates two important norms in coseismic fault behavior. First, peak net slip of ~11.5 m and mean net slip of ~8.3 m on the main strand far exceed values expected from empirical slip-to-length relationships (*9*). As discussed by Langridge *et al.* (*28*), this observation alone has important repercussions for interpretations of paleoseismic data and for seismic hazard studies, particularly within regions of diffuse faulting like the Marlborough Fault System, where multifault ruptures may be common. Second, vertical offsets of ~6 to 9.5 m along the northern Papatea fault, where average dip exceeds 70°, contradict the theory of Andersonian mechanics, which supposes reverse faults usually occur with shallow dips of ~30° (*36*).

### Anelastic surface deformation

**Fig. 5:**
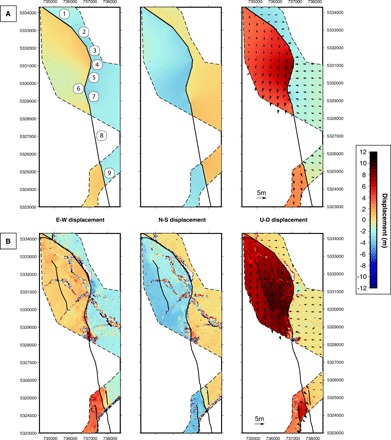
Comparison of lidar-derived 3D displacement field to elastic-modeled surface deformation around the Papatea fault. (Left) E-W, (middle) N-S, and (right) up-down (**A**) elastic model and (**B**) observed surface displacement fields around the Papatea fault. In (A), we use nine rectangular dislocations (numbered in white circles) embedded in an elastic half-space (*14*) to a depth of 10 km, imposing values of dip, rake, and slip that are representative of our lidar profiling measurements (see table S2 for model inputs). The black lines show the model fault trace, and dashed black lines show the extent of the double lidar coverage. The black lines in (B) show the mapped fault traces. Black vectors overlying the up-down displacement fields indicate horizontal displacements calculated using a block mean of dimension 400 m by 400 m.

Hamling *et al.* (*4*) used rectangular dislocations within an elastic half-space to invert InSAR data for coseismic slip in the Kaikōura earthquake but could not fit surface displacements surrounding the Papatea fault with this model. We reexamined this using simple forward models of the Papatea fault by simplifying its curvilinear surface trace with nine connected rectangular dislocations, each extended to 10-km depth within an elastic half-space (*37*) and imposing representative values of fault slip, dip, and rake on each segment. Like Hamling *et al.* (*4*), we find that the simple elastic model fits the lidar data very poorly, especially in the vertical displacement direction, where the model predicts much smaller hanging wall uplift but much larger footwall subsidence than the data indicate (Fig. 5). We also tested the influence of forward modeling slip along (i) a low-angle extension of the steep surface Papatea fault (for an overall listric geometry; fig. S3), (ii) the Jordan and Kekerengu faults (fig. S4), and (iii) the underlying subduction interface (fig. S5). We based the geometries and slip values of these additional, neighboring faults on published Kaikōura earthquake rupture models (*4*, *6*, *7*, *22*–*26*, *33*). However, the discrepancies between the observed and forward modeled surface deformation fields still could not be reconciled.

**Fig. 6:**
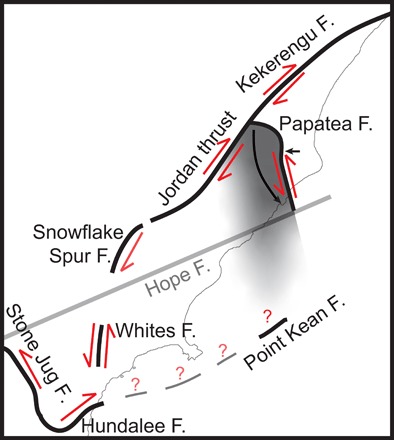
Schematic diagram of the Papatea block and neighboring ruptures within the large step-over of the Kaikōura earthquake. Solid black lines indicate approximate mapped faults (*8*, *24*, *28*). Dashed gray lines indicate possible fault rupture locations (*21*). The black arrows near the Papatea fault demonstrate the observed horizontal displacement. The gray shaded region represents the area of the large vertical uplift that was forced to “pop up” during the earthquake due to a localized space problem caused by neighboring ruptures.

We conclude that the Papatea fault ruptured anelastically, by which we mean that the highly asymmetric displacements on either side of the fault cannot be fit by elastic dislocation models. We do not consider this the result of a pronounced material contrast across the fault, for which there is no geological evidence (*28*, *29*). We suggest that the Papatea fault did not release built-up interseismic strain energy as is conventionally assumed. Instead, the exceedingly large uplift of the Papatea block was forced by neighboring, elastic fault ruptures, introducing a space problem within the major step-over in the Kaikōura earthquake (Fig. 6).

A broader implication is that in certain circumstances, dynamic earthquakes can activate faults without apparent accumulated strain energy. This supports inferences that creeping fault segments, which like the Papatea fault lack stored strain, may sometimes rupture coseismically, under the influence of an approaching high-energy earthquake (*38*). Ruptures such as these may also explain why some faults exhibit low geodetic strain accumulation rates but much larger geological slip rates (*39*). Therefore, we cannot rely on strain accumulation rates alone as an indicator for rupture potential. This has important implications for seismic hazard because these types of activated ruptures may produce multimeter slip, as the Papatea rupture demonstrates.

## CONCLUSIONS

We reveal that (i) the Papatea fault twists from moderate dip angles in the south to subvertical in the north, where multimeter vertical slip defies Andersonian fault mechanics, and (ii) its deformation field cannot be fit using elastic models. We suggest that the Papatea fault did not release stored elastic energy in the manner normally assumed, perhaps explaining why the fault appears quiescent in many back-projection models [e.g., (*7*, *15*, *16*)]. Instead, these highly unusual kinematics indicate a reactivated structure that slipped anelastically to accommodate strain release from surrounding elastic ruptures during the cascading 2016 Kaikōura earthquake. These results suggest that the common practice of modeling earthquakes as slip on planar faults embedded in an elastic half-space may not always be appropriate. Furthermore, the Papatea rupture demonstrates that faults without stored strain energy still have the potential to generate multimeter slip in large earthquakes.

## MATERIALS AND METHODS

### 3D surface displacements from differential lidar and ICP

We analyzed deformation within the Papatea fault zone using 3D differential lidar surface displacement fields sourced from pre- and postevent airborne lidar surveys collected in July 2012 and November 2016 to January 2017 [see Clark *et al.* (*21*) for further details of these datasets]. The double coverage captures most of the onshore Papatea fault, with only a short middle section and the northernmost intersection with the Jordan and Kekerengu faults missing. With no other major earthquakes occurring within this time frame in this area, differencing the pre- and postevent lidar data captured the coseismic deformation of the 2016 Kaikōura earthquake. This repeat lidar dataset was previously analyzed by Langridge *et al.* (*28*), and the coastal part by Clark *et al.* (*21*), using differenced DEMs. The resulting elevation change maps provide a high spatial resolution measure of vertical deformation but are subject to biases from the unconstrained horizontal components of the displacement field, which are likely to be substantial in this area.

To overcome this problem, we used the ICP algorithm to align square subsets (“cells”) of the pre-event lidar point cloud to equivalent subsets of the postevent point cloud (*11*). This retrieved the 3D rigid body transformation (translations and rotations around Cartesian axes) that minimized average closest point distances between each pair of cells. Using the provided point classifications, we first stripped each point cloud of all vegetation, building, and water returns, leaving only the bare earth surface. For the ICP alignment, we implemented a “sparse ICP” approach (*12*) with point-to-plane alignment within the MATLAB source code that is made available as a supplement. This approach uses sparsity-inducing norms that unify various criteria for downweighting outliers, data gaps, and geometric boundaries (*13*) under a single parameter *p*. This parameter is the exponent of the sparse norm with a value between 0 and 1. This technique allowed for automatic classification of outliers and inliers, where the latter were given greater prominence in the next iterative step, unlike traditional ICP methods that do not distinguish between outliers and inliers. Overall, this approach yielded a robust algorithm that reduced the impact of outliers and data gaps, which would otherwise lead iterative registration toward erroneous local, rather than global, minima (*12*). After experimentation, we used a 50-m cell size with an additional 10-m border around the postevent cells to capture any lateral shift of topography in the earthquake (*40*). We used a sliding window to enhance the spatial resolution further, for a final displacement grid resolution of 25 m.

The resulting Cartesian *x*, *y*, and *z* axis transformations represent local surface displacements in the east-west, north-south, and up-down directions, respectively (Fig. 2B). Deformation is coherent along many of the Papatea fault surface ruptures, which are clearly expressed as sharp displacement discontinuities in the displacement fields. However, the Back-basin and Corner Hill faults (*28*) did not show clear discontinuities and are not included in our analysis. Coherence is lost in a few areas, most notably where land sliding or reorganization of the Clarence River have wholly altered the shape of the topographic cells, rendering the scattered ICP alignments meaningless. Vertical displacements are smoother (less noisy) than horizontal displacements, in common with other earthquake surface displacement fields mapped using ICP (*14*), reflecting the fact that topographic ground returns are most closely spaced in the *z* dimension. In comparison, Hamling *et al.*’s (*4*) 3D displacement field derived from the SAR interferometry and pixel offset measurements is an order of magnitude coarser (~330 m × 450 m) and appears highly pixelated at the resolution of the lidar results (Fig. 2, A and B). Colocated SAR- and lidar-derived displacements show multimeter-level scatter but no systematic offset in either of the *x*, *y*, or *z* displacement components (fig. S1). Visually, the lidar displacements appear in reasonable agreement with those derived from differential photogrammetry (*24*), although we are unable to make a statistical comparison between the two.

The fine detail evident in the lidar data proved crucial in being able to profile the displacement field, measure fault offsets, and, thus, resolve fault dip. This was especially the case along the coastal section of the Papatea fault, where we observe all three displacement components changing over short, subkilometer distances on three closely spaced fault strands.

### Fault slip vector, dip, and rake measurements

For our detailed analysis of the Papatea fault geometry and kinematics, we adopted and modified an approach developed by Lajoie *et al.* (*14*) in their analysis of the 2010 El Mayor-Cucapah (Mexico) earthquake. The first step was to map rupture traces directly from the clear discontinuities in surface displacements. Next, fault-perpendicular swath profiles were extracted through each of the *x*, *y*, and *z* displacement fields at regular intervals along each rupture trace [examples are given in Fig. 2 (C to H)]. Coseismic fault offsets were measured from each profile by calculating least squares linear fits to displacement data points on each side of the fault damage zone, extrapolating these trends to the fault, and differencing them. This procedure offers three key advantages over geological methods of measuring fault offsets from single postearthquake surveys: (i) There is no assumption that the data trends on either side of the fault represent surfaces of the same age; (ii) there is no need to assume initial surface or landform geometries; and (iii) offset measurements are no longer prone to biases arising from landform geometry. Offset uncertainties were estimated by extrapolation and differencing of the 50% confidence bounds. After testing, we used swath profiles with lengths of 900 m and widths of 100 m to ensure that coherent ground displacements were captured on either side of the fault, given the presence of noise (e.g., landslides, Clarence River) and distributed deformation within the fault damage zone. In practice, only a subset of profiles could be used in subsequent measurements due to the variable quality of the deformation field.

At each measurement point, the *x*, *y*, and *z* offsets together describe the Cartesian fault slip vector, which by definition must lie in the plane of the fault. Paired with the local fault strike measured at the start of the procedure, this slip vector thus defines a unique fault dip and rake. [An exception would arise where the slip vector horizontal and parallel to the fault trace, as for a pure strike-slip fault—see Lajoie *et al.* (*14*) for a discussion—but for the oblique Papatea fault, this is never the case.] In an advance on the procedure outlined by Lajoie *et al.* (*14*), we also propagated estimated uncertainties in the *x*, *y*, and *z* offsets into uncertainties in fault dip. We did so using a Monte Carlo simulation, calculating dip 100 times using random draws from Gaussian distributions in the *x*, *y*, and *z* offsets, and determining SD values of the resulting dip distribution. In this way, and where the lidar coverage and quality permit it, we were able to determine the vertical, lateral, and fault normal components of slip, the net slip, the fault dip, the rake, and the statistical uncertainties in these parameters at closely spaced intervals along each of the four strands of the Papatea fault (table S1).

## Supplementary Material

http://advances.sciencemag.org/cgi/content/full/5/10/eaax5703/DC1

Download PDF

Sparse ICP code and documentation

Unusual kinematics of the Papatea fault (2016 Kaikōura earthquake) suggest anelastic rupture

## SUPPLEMENTARY MATERIALS

Supplementary material for this article is available at http://advances.sciencemag.org/cgi/content/full/5/10/eaax5703/DC1 Fig. S1. SAR- and lidar-derived ground displacements. Fig. S2. Kinematics of the coastal Papatea fault zone. Fig. S3. Comparison of lidar-derived 3D displacement field to elastic modeled surface deformation around the main strand surface rupture with listric structure below. Fig. S4. Comparison of lidar-derived 3D displacement field to elastic modeled surface deformation around the main strand surface rupture with Jordan and Kekerengu ruptures. Fig. S5. Comparison of lidar-derived 3D displacement field to elastic modeled surface deformation around the main strand surface rupture with plate interface below. Table S1. Kinematic parameters from rupture profiling. Table S2. Elastic forward model parameters to produce figs. S3 to S5. Sparse ICP code and documentation

## References

[1] Meng L., Ampuero J.-P., Stock J., Duputel Z., Luo Y., Tsai V. C., Earthquake in a maze: Compressional rupture branching during the 2012 *M*_w_ 8.6 Sumatra earthquake. Science 337, 724–726 (2012). 22821986 10.1126/science.1224030

[2] Fletcher J. M., Oskin M. E., Teran O. J., The role of a keystone fault in triggering the complex El Mayor–Cucapah earthquake rupture. Nat. Geosci. 9, 303–307 (2016).

[3] Nissen E., Elliott J. R., Sloan R. A., Craig T. J., Funning G. J., Hutko A., Parsons B. E., Wright T. J., Limitations of rupture forecasting exposed by instantaneously triggered earthquake doublet. Nat. Geosci. 9, 330–336 (2016).

[4] Hamling I. J., Hreinsdóttir S., Clark K., Elliott J., Liang C., Fielding E., Litchfield N., Villamor P., Wallace L., Wright T. J., D’Anastasio E., Bannister S., Burbidge D., Denys P., Gentle P., Howarth J., Mueller C., Palmer N., Pearson C., Power W., Barnes P., Barrell D. J. A., Van Dissen R., Langridge R., Little T., Nicol A., Pettinga J., Rowland J., Stirling M., Complex multifault rupture during the 2016 *M*_w_ 7.8 Kaikōura earthquake, New Zealand. Science 356, eaam7194 (2017). 28336563 10.1126/science.aam7194

[5] Lamb S., Arnold R., Moore J. D. P., Locking on a megathrust as a cause of distributed faulting and fault-jumping earthquakes. Nat. Geosci. 11, 871–875 (2018).

[6] Hollingsworth J., Ye L., Avouac J.-P., Dynamically triggered slip on a splay fault in the *M_w_* 7.8, 2016 Kaikoura (New Zealand) earthquake. Geophys. Res. Lett. 44, 3517–3525 (2017).

[7] Zhang H., Koper K. D., Pankow K., Ge Z., Imaging the 2016 *M_w_* 7.8 Kaikoura, New Zealand, earthquake with teleseismic *P* waves: A cascading rupture across multiple faults. Geophys. Res. Lett. 44, 4790–4798 (2017).

[8] Litchfield N., Villamor P., Van Dissen R. J., Nicol A., Barnes P. M., Barrell D. J. A., Pettinga J. R., Langridge R. M., Little T. A., Mountjoy J. J., Ries W. F., Rowland J., Fenton C., Stirling M. W., Kearse J., Berryman K. R., Cochran U. A., Clark K. J., Hemphill-Haley M., Khajavi N., Jones K. E., Archibald G., Upton P., Asher C., Benson A., Cox S. C., Gasston C., Hale D., Hall B., Hatem A. E., Heron D. W., Howarth J., Kane T. J., Lamarche G., Lawson S., Lukovic B., McColl S. T., Madugo C., Manousakis J., Noble D., Pedley K., Sauer K., Stahl T., Strong D. T., Townsend D. B., Toy V., Williams J., Woelz S., Zinke R., Surface rupture of multiple crustal faults in the 2016 *M*_w_ 7.8 Kaikōura, New Zealand, Earthquake. Bull. Seismol. Soc. Am. 108, 1496–1520 (2018).

[9] Wells D. L., Coppersmith K. J., New empirical relationships among magnitude, rupture length, rupture width, rupture area, and surface displacement. Bull. Seismol. Soc. Am. 84, 974–1002 (1994).

[10] Kaiser A., Balfour N., Fry B., Holden C., Litchfield N., Gerstenberger M., D’Anastasio E., Horspool N., McVerry G., Ristau J., Bannister S., Christophersen A., Clark K., Power W., Rhoades D., Massey C., Hamling I., Wallace L., Mountjoy J., Kaneko Y., Benites R., van Houtte C., Dellow S., Wotherspoon L., Elwood K., Gledhill K., The 2016 Kaikōura, New Zealand, Earthquake: Preliminary seismological report. Seismol. Res. Lett. 88, 727–739 (2017).

[11] Chen Y., Medioni G., Object modeling by registration of multiple range images. Image Vis. Comp. 10, 2724–2729 (1991).

[12] Bouaziz S., Tagliasacchi A., Pauly M., Sparse iterative closest point. Comp. Graph. Forum 32, 113–123 (2013).

[13] Rusinkiewicz S., Levoy M., Efficient variants of the ICP algorithm. 3dim 1, 145–152 (2001).

[14] Lajoie L. J., Nissen E., Johnson K. L., Arrowsmith J. R., Glennie C. L., Hinojosa-Corona A., Oskin M. E., Extent of low-angle normal slip in the 2010 El Mayor-Cucapah (Mexico) earthquake from differential lidar. J. Geophys. Res. 124, 943–956 (2019).

[15] Tan F., Ge Z., Kao H., Nissen E., Validation of the 3-D phase-weighted relative back projection technique and its application to the 2016 *M*_w_ 7.8 Kaikōura Earthquake. Geophys. J. Int. 217, 375–388 (2019).

[16] Wang D., Chen Y., Wang Q., Mori J., Complex rupture of the 13 November 2016 *M*_w_ 7.8 Kaikoura, New Zealand earthquake: Comparison of high-frequency and low-frequency observations. Tectonophysics 733, 100–107 (2018).

[17] Litchfield N. J., Van Dissen R., Sutherland R., Barnes P. M., Cox S. C., Norris R., Beavan R. J., Langridge R., Villamor P., Berryman K., Stirling M., Nicol A., Nodder S., Lamarche G., Barrell D. J. A., Pettinga J. R., Little T., Pondard N., Mountjoy J. J., Clark K., A model of active faulting in New Zealand. N. Z. J. Geol. Geophys. 57, 32–56 (2013).

[18] Wallace L. M., Beavan J., McCaffrey R., Berryman K., Denys P., Balancing the plate motion budget in the South Island, New Zealand using GPS, geological and seismological data. Geophys. J. Int. 168, 332–352 (2007).

[19] Beavan J., Wallace L. M., Palmer N., Denys P., Ellis S., Fournier N., Hreinsdottir S., Pearson C., Denham M., New Zealand GPS velocity field: 1995–2013. N.Z. J. Geol. Geophys. 59, 5–14 (2016).

[20] Mason D. P. M., Little T. A., Refined slip distribution and moment magnitude of the 1848 Marlborough earthquake, Awatere Fault, New Zealand. N.Z. J. Geol. Geophys. 49, 375–382 (2006).

[21] Clark K. J., Nissen E. K., Howarth J. D., Hamling I. J., Mountjoy J. J., Ries W. F., Jones K., Goldstien S., Cochran U. A., Villamor P., Hreinsdóttir S., Litchfield N. J., Mueller C., Berryman K. R., Strong D. T., Highly variable coastal deformation in the 2016 M_w_ 7.8 Kaikōura earthquake reflects rupture complexity along a transpressional plate boundary. Earth Planet. Sci. Lett. 474, 334–344 (2017).

[22] Bai Y., Lay T., Cheung K. F., Ye L., Two regions of seafloor deformation generated the tsunami for the 13 November 2016, Kaikoura, New Zealand earthquake. Geophys. Res. Lett. 44, 6597–6606 (2017).

[23] Xu W., Feng G., Meng L., Zhang A., Ampuero J. P., Bürgmann R., Fang L., Transpressional rupture cascade of the 2016 M_w_ 7.8 Kaikoura Earthquake, New Zealand. J. Geophys. Res. Solid Earth 123, 2396–2409 (2018).

[24] Zinke R., Hollingsworth J., Dolan J. F., Van Dissen R., 3D surface deformation in the 2016 M_w_ 7.8 Kaikōura, New Zealand earthquake from optical image correlation: Implications for strain localization and long–term evolution of the Pacific–Australian plate boundary. Geochem. Geophys. Geosyst. 20, 1609–1628 (2019).

[25] Holden C., Kaneko Y., D'Anastasio E., Benites R., Fry B., Hamling I. J., The 2016 Kaikōura Earthquake revealed by kinematic source inversion and seismic wavefield simulations: Slow rupture propagation on a geometrically complex crustal fault network. Geophys. Res. Lett. 44, 11320–11328 (2017).

[26] Duputel Z., Rivera L., Long-period analysis of the 2016 Kaikoura earthquake. Phys. Earth Planet. Inter. 265, 62–66 (2017).

[27] Wang T., Wei S., Shi X., Qiu Q., Li L., Peng D., Weldon R. J., Barbot S., The 2016 Kaikōura Earthquake: Simultaneous rupture of the subduction interface and overlying faults. Earth Planet. Sci. Lett. 482, 44–51 (2018).

[28] Langridge R. M., Rowland J., Villamor P., Mountjoy J., Townsend D. B., Nissen E., Madugo C., Ries W. F., Gasston C., Canva A., Hatem A. E., Hamling I., Coseismic rupture and preliminary slip estimates for the Papatea fault and its role in the 2016 *M*_w_ 7.8 Kaikōura, New Zealand, Earthquake. Bull. Seismol. Soc. Am. 108, 1596–1622 (2018).

[29] M. Rattenbury, D. Townsend, M. Johnston, *Geology of the Kaikoura Area: Scale 1:250,000 Geological Map* (GNS Science, Lower Hutt, 2006).

[30] Langridge R. M., Ries W. F., Litchfield N. J., Villamor P., van Dissen R., Barrell D. J. A., Rattenbury M. S., Heron D. W., Haubrock S., Townsend D. B., Lee J. M., Berryman K. R., Nicol A., Cox S. C., Stirling M. W., The New Zealand active faults database. N.Z. J. Geol. Geophys. 59, 86–96 (2016).

[31] Klinger Y., Okubo K., Vallage A., Champenois J., Delorme A., Rougier E., Lei Z., Knight E. E., Munjiza A., Satriano C., Baize S., Langridge R., Bhat H. S., Earthquake damage patterns resolve complex rupture processes. Geophys. Res. Lett. 45, 10279–10287 (2018).

[32] Ulrich T., Gabriel A.-A., Ampuero J.-P., Xu W., Dynamic viability of the 2016 Mw 7.8 Kaikoura earthquake cascade on weak crustal faults. Nat. Commun. 10, 1213 (2019). 30872591 10.1038/s41467-019-09125-w PMC6418120

[33] Wen Y.-Y., Ma K.-F., Fry B., Multiple-fault, slow rupture of the 2016 M_w_ 7.8 Kaikōura, New Zealand, earthquake: Complementary insights from teleseismic and geodetic data. Bull. Seismol. Soc. Am. 108, 1774–1783 (2018).

[34] Morishita Y., Kobayashi T., Fujiwara S., Yarai H., Complex crustal deformation of the 2016 Kaikōura, New Zealand, earthquake revealed by ALOS-2. Bull. Seismol. Soc. Am. 107, 2676–2686 (2017).

[35] Mackenzie D., Elliott A. J., Untangling tectonic slip from the potentially misleading effects of landform geometry. Geosphere 13, 1310–1328 (2017).

[36] E. M. Anderson, *The Dynamics of Faulting and Dyke Formation with Applications to Britain* (Oliver and Boyd, ed. 2, 1951), 206 p.

[37] Okada Y., Surface deformation due to shear and tensile faults in a half-space. Bull. Seismol. Soc. Am. 75, 1135–1154 (1985).

[38] Noda H., Lapusta N., Stable creeping fault segments can become destructive as a result of dynamic weakening. Nature 493, 518–521 (2013). 23302798 10.1038/nature11703

[39] Chuang R. Y., Johnson K. M., Reconciling geologic and geodetic model fault slip-rate discrepancies in Southern California: Consideration of nonsteady mantle flow and lower crustal fault creep. Geology 39, 627–630 (2011).

[40] Nissen E., Krishnan A. K., Arrowsmith J. R., Saripalli S., Three-dimensional surface displacements and rotations from differencing pre-and post-earthquake LiDAR point clouds. Geophys. Res. Lett. 39, L16301 (2012).

