# Supplementary material for: Unusual kinematics of the Papatea fault (2016 Kaikōura earthquake) suggest anelastic rupture
Source: Sci Adv. 2019 Oct 2;5(10):eaax5703. doi: 10.1126/sciadv.aax5703 (PMC6774718; doi:10.1126/sciadv.aax5703)
Supplement: http://advances.sciencemag.org/cgi/content/full/5/10/eaax5703/DC1 [file supp_5_10_eaax5703__index.html]

Science Advances | Science AdvancesAAASSearchScience AdvancesMenu

## Supplementary Materials

**The PDF file includes:**

- Fig. S1. SAR- and lidar-derived ground displacements.
- Fig. S2. Kinematics of the coastal Papatea fault zone.
- Fig. S3. Comparison of lidar-derived 3D displacement field to elastic modeled surface deformation around the main strand surface rupture with listric structure below.
- Fig. S4. Comparison of lidar-derived 3D displacement field to elastic modeled surface deformation around the main strand surface rupture with Jordan and Kekerengu ruptures.
- Fig. S5. Comparison of lidar-derived 3D displacement field to elastic modeled surface deformation around the main strand surface rupture with plate interface below.
- Table S1. Kinematic parameters from rupture profiling.
- Table S2. Elastic forward model parameters to produce figs. S3 to S5.

Download PDF

**Other Supplementary Material for this manuscript includes the following:**

- Sparse ICP code and documentation (.tar format)

**Files in this Data Supplement:**

- Adobe PDF - aax5703\_SM.pdf
