# Supplementary material for: Unusual kinematics of the Papatea fault (2016 Kaikōura earthquake) suggest anelastic rupture
Source: Sci Adv. 2019 Oct 2;5(10):eaax5703. doi: 10.1126/sciadv.aax5703 (PMC6774718; doi:10.1126/sciadv.aax5703)
Supplement: Download PDF [file aax5703_SM.pdf]

Science Advances
AAAS

advances.sciencemag.org/cgi/content/full/5/10/eaax5703/DC1

Supplementary Materials for

Unusual kinematics of the Papatea fault (2016 Kaikōura earthquake) suggest anelastic rupture

A. Diederichs*, E. K. Nissen, L. J. Lajoie, R. M. Langridge, S. R. Malireddi, K. J. Clark, I. J. Hamling, A. Tagliasacchi

*Corresponding author. Email: adiederi@uvic.ca

Published 2 October 2019, *Sci. Adv.* **5**, eaax5703 (2019)
DOI: 10.1126/sciadv.aax5703

The PDF file includes:

Fig. S1. SAR- and lidar-derived ground displacements.
Fig. S2. Kinematics of the coastal Papatea fault zone.
Fig. S3. Comparison of lidar-derived 3D displacement field to elastic modeled surface deformation around the main strand surface rupture with listric structure below.
Fig. S4. Comparison of lidar-derived 3D displacement field to elastic modeled surface deformation around the main strand surface rupture with Jordan and Kekerengu ruptures.
Fig. S5. Comparison of lidar-derived 3D displacement field to elastic modeled surface deformation around the main strand surface rupture with plate interface below.
Table S1. Kinematic parameters from rupture profiling.
Table S2. Elastic forward model parameters to produce figs. S3 to S5.

Other Supplementary Material for this manuscript includes the following:

(available at advances.sciencemag.org/cgi/content/full/5/10/eaax5703/DC1)

Sparse ICP code and documentation (.tar format)

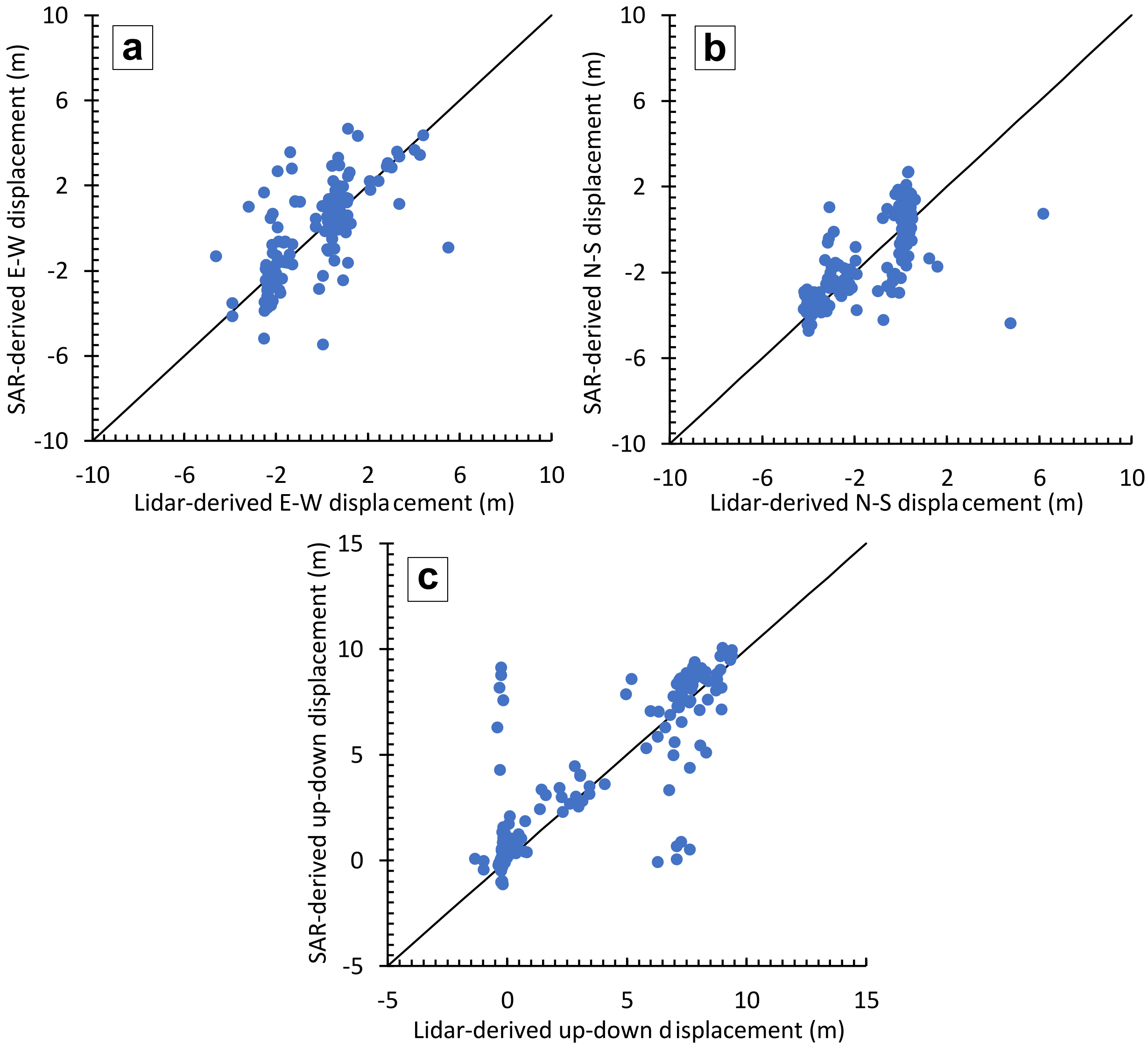

Fig. S1. SAR- and lidar-derived ground displacements. Scatter plots of SAR-derived displacements from Hamling et al[4]. (vertical axes) and block median differential lidar displacements (horizontal axes), in the (**a**) E–W, (**b**) N–S, and (**c**) vertical axis directions. The differential lidar block median values were calculated at the pixel resolution of the SAR-derived displacement field.

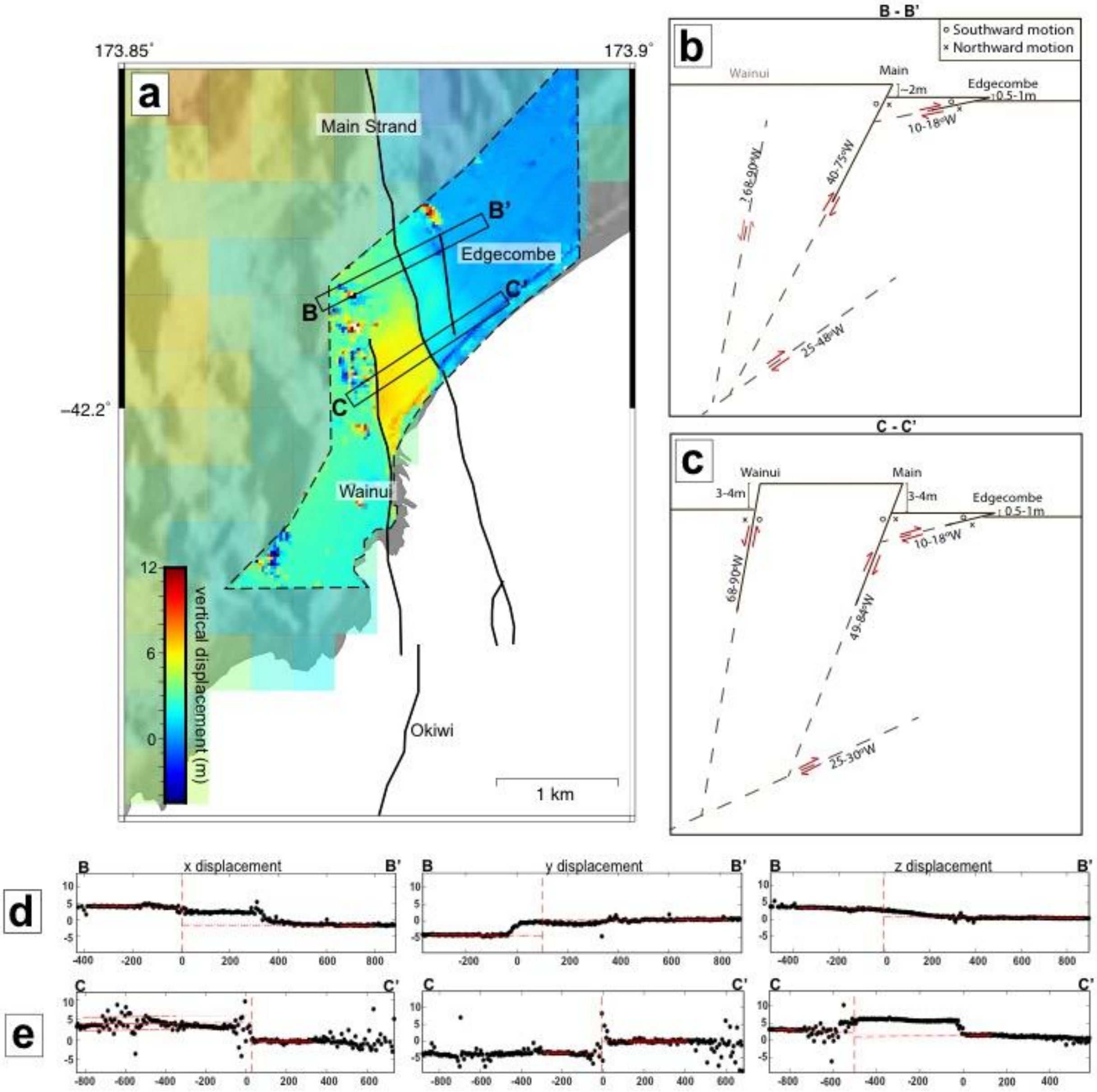

Fig. S2. Kinematics of the coastal Papatea fault zone. (**a**) Differential lidar vertical displacements in the coastal section of the Papatea fault zone. Outside the lidar double coverage area, marked by the dashed black line, vertical displacements are from Hamling et al[4]. Fault-perpendicular swath profiles used to derive dips in panels (**b**) and (**c**) are shown by black rectangles B-B' and C-C'. (b) Schematic cross section B-B' portraying both the shallow dip of individual strands and deeper fault dip, estimated from far-field offset extrapolated to the main strand. Strike-slip components are represented by open circles (southward motion) and crosses (northward motion). (c) Schematic cross section C-C', estimated as in (b). (**d-e**) Sample profile swaths through the x (left), y (middle) and z (right) lidar surface displacement fields that correspond to B-B' (d) and C-C' (e).

Structural Interpretation of the Coastal Papatea Fault Zone (fig. S2)

At the coast, the main strand is straddled to the west by the Wainui trace - which may merge offshore with the Okiwi trace - and to the east by the shorter Edgecombe trace (fig. S2a). All three faults are W-dipping, with progressively steeper dips from east (Edgecombe) to west (Wainui), such that the faults likely converge or merge at depth. To estimate the dip of the underlying master structure, we determined cumulative fault zone offsets along 100 m interval profiles by extrapolating far-field displacements from outside of the fault zone to the central fault (fig. S2d-e). Resulting dip values of ~25-48º W are considerably gentler than those of the northern Papatea fault zone, ~5 km to the north. Schematic cross sections along two profiles imply significant complexity in fault geometry and kinematics, likely reflecting the step-over and transferal of strain between the western (offshore Okiwi, and Wainui) strand and the main strand (fig. S2b-c).

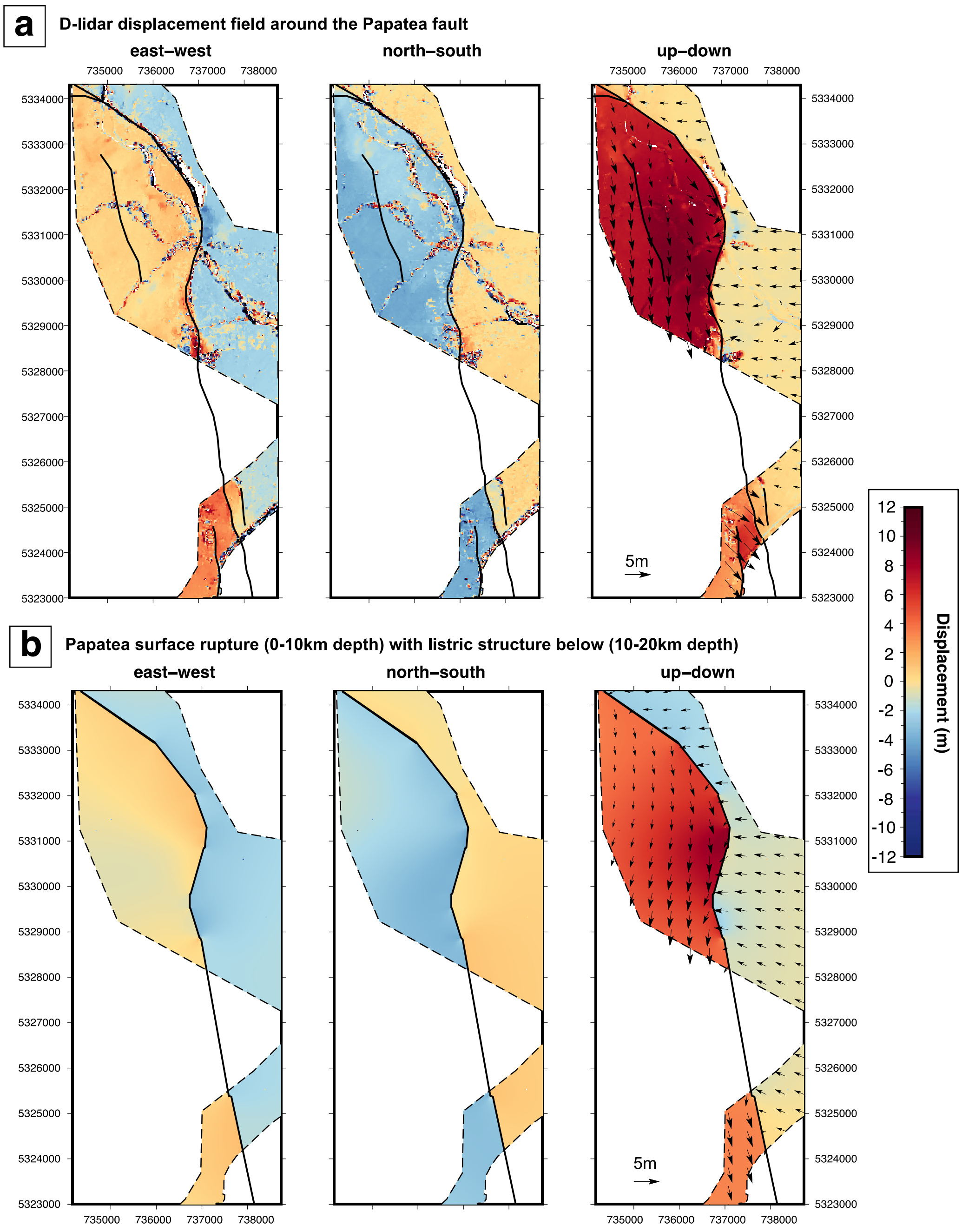

Fig. S3. Comparison of lidar-derived 3D displacement field to elastic modeled surface deformation around the main strand surface rupture with listric structure below. (Left) E-W, (middle) N-S, and (right) up-down surface displacement fields observed from **(a)** differential lidar and **(b)** an elastic forward model of the main strand rupture together with a gently-dipping rupture below it, at 10 – 20 km depth. Dashed black lines showing the extent of the double lidar coverage and black vectors overlying the up-down displacement fields indicate horizontal displacements calculated using a block mean of dimension 400 x 400 m. Black lines in (a) are mapped fault traces. Model parameters for (b) are tabulated in table S2.

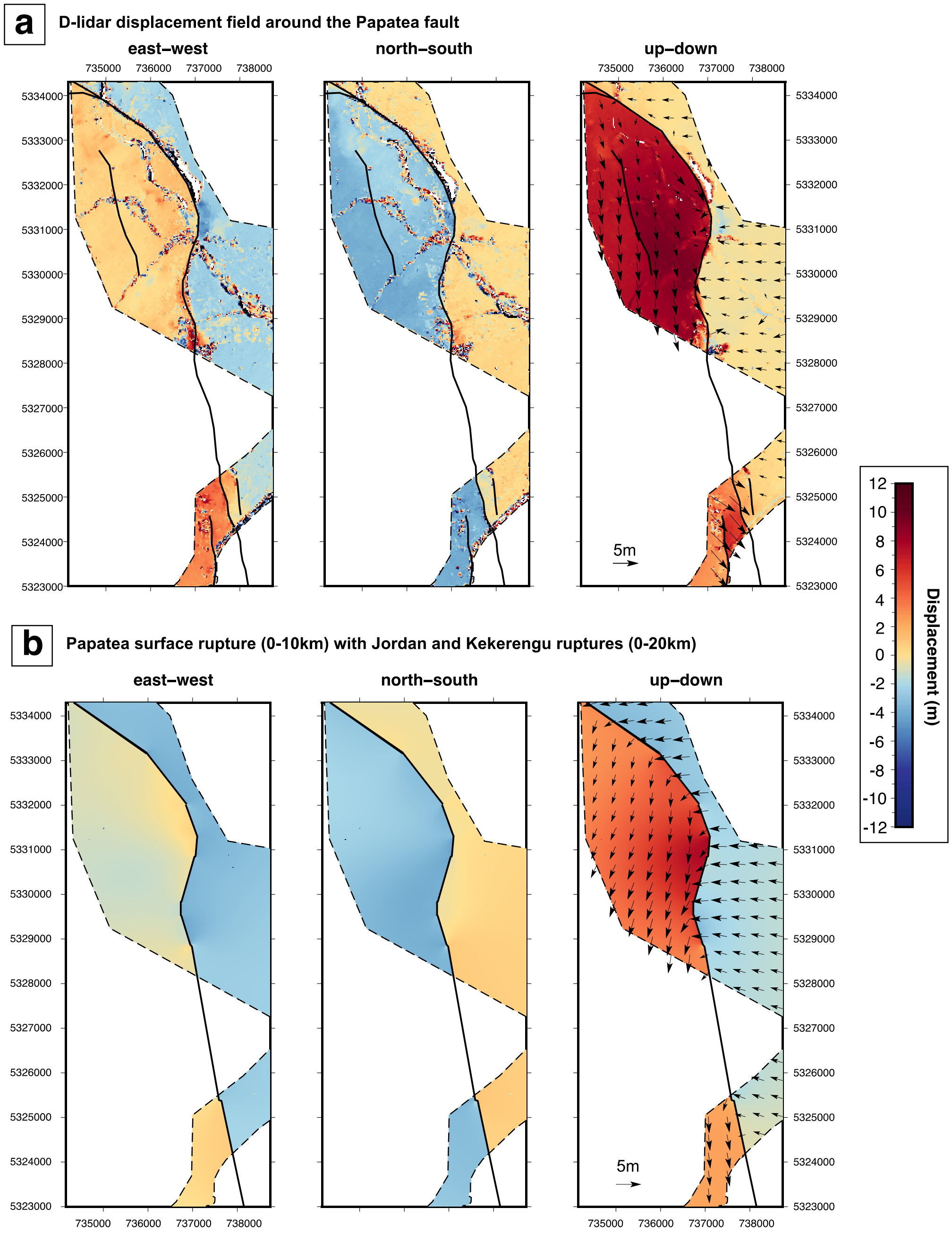

Fig. S4. Comparison of lidar-derived 3D displacement field to elastic modeled surface deformation around the main strand surface rupture with Jordan and Kekerengu ruptures. (Left) E-W, (middle) N-S, and (right) up-down surface displacement fields observed from (**a**) differential lidar and (**b**) an elastic forward model of the main strand rupture together with the Jordan and Kekerengu fault ruptures. Dashed black lines showing the extent of the double lidar coverage and black vectors overlying the up-down displacement fields indicate horizontal displacements calculated using a block mean of dimension 400 x 400 m. Black lines in (a) are mapped fault traces. Model parameters for (b) are tabulated in table S2.

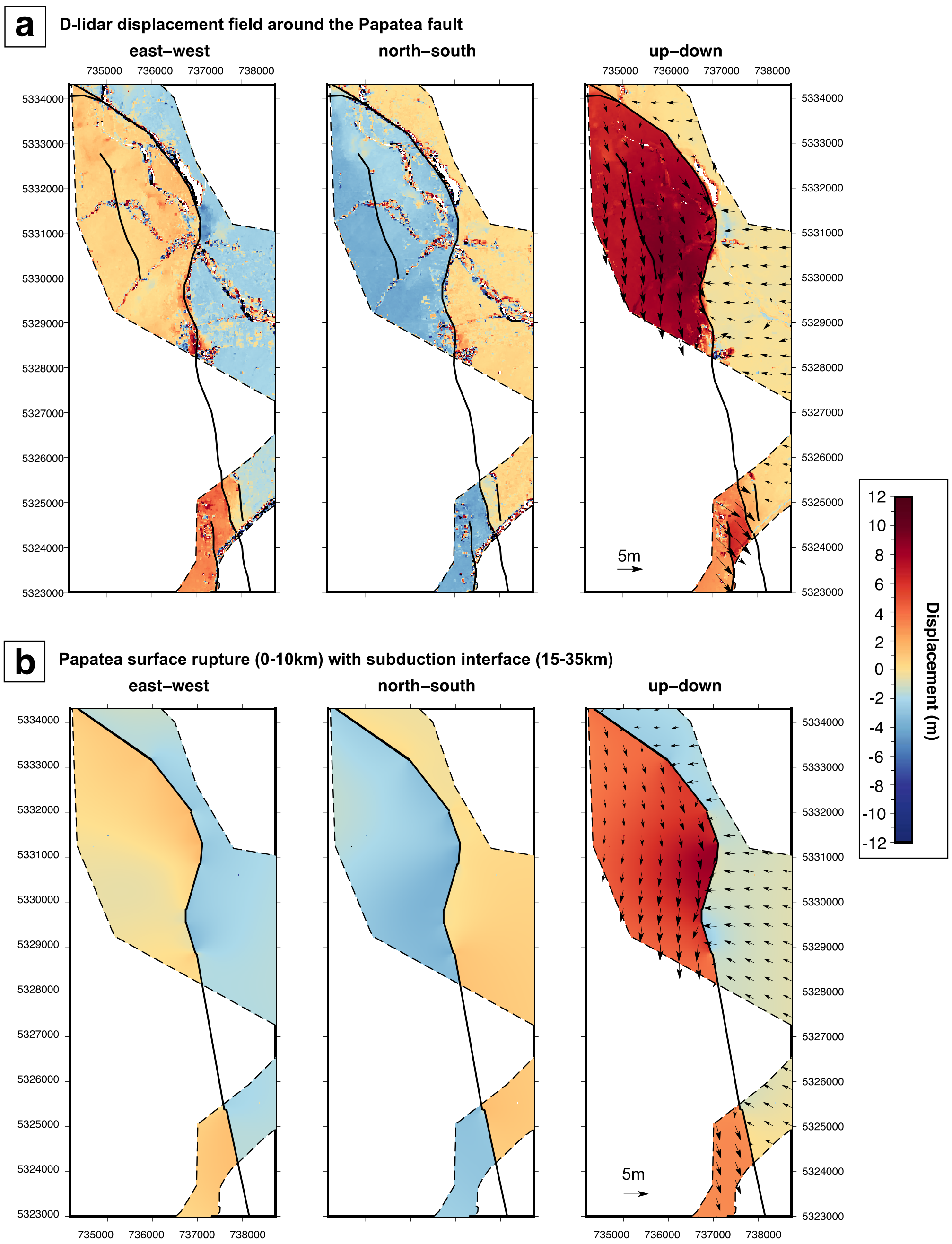

Fig. S5. Comparison of lidar-derived 3D displacement field to elastic modeled surface deformation around the main strand surface rupture with plate interface below. (Left) E-W, (middle) N-S, and (right) up-down surface displacement fields observed from (**a**) differential lidar and (**b**) an elastic forward model of the main strand rupture together with 2m of slip on the subduction interface below. Dashed black lines showing the extent of the double lidar coverage and black vectors overlying the up-down displacement fields indicate horizontal displacements calculated using a block mean of dimension 400 x 400 m. Black lines in (a) are mapped fault traces. Model parameters for (b) are tabulated in table S2.

Table S1. Kinematic parameters from rupture profiling. Parameters calculated from fault-perpendicular swath profiles along the (M)ain, (Wh)arekiri, (Wa)inui and (E)dgecombe strands, as well as (F)ar field displacements across all three coastal faults. Latitude and longitude refer to profile center-points, listed from N to S for each strand. *x*, *y* and *z* offsets refer to motion of the west side of the fault with respect to the east side.

label	Latitude	Longitude	strike	x offset (m)	y offset (m)	z offset (m)	net offset (m)	fault dip	rake
Wharekiri strand									
Wh 1	-42.12520°	173.84372°	140.5°	0.20 ± 0.20	-0.69 ± 0.15	-0.70 ± 0.18	1.00 ± 0.31	67.7° SW ± 5.2°	-48.8°
Wh 2	-42.12692°	173.84430°	350.9°	0.30 ± 0.28	-1.13 ± 0.17	-0.77 ± 0.14	1.40 ± 0.36	81.0° E ± 3.3°	33.9°
Wh 3	-42.12781°	173.84450°	350.9°	0.41 ± 0.27	-0.90 ± 0.17	-0.50 ± 0.12	1.11 ± 0.34	62.4° E ± 3.7°	30.9°
Wh 4	-42.12869°	173.84470°	350.9°	0.51 ± 0.31	-0.99 ± 0.20	-0.72 ± 0.19	1.32 ± 0.41	64.4° E ± 4.3°	37.2°
Wh 5	-42.12958°	173.84494°	344.2°	0.73 ± 0.29	-1.29 ± 0.37	-0.92 ± 0.17	1.74 ± 0.50	68.9° E ± 4.3°	34.4°
Wh 6	-42.13395°	173.84638°	349.2°	0.42 ± 0.26	-0.83 ± 0.21	-1.22 ± 0.12	1.53 ± 0.35	77.9° E ± 3.1°	54.5°
Wh 7	-42.13483°	173.84662°	349.2°	0.52 ± 0.14	-1.04 ± 0.08	-1.34 ± 0.07	1.77 ± 0.18	76.7° E ± 1.3°	50.8°
Wh 8	-42.13651°	173.84745°	338.3°	0.48 ± 0.09	-0.97 ± 0.18	-1.51 ± 0.10	1.86 ± 0.22	86.6° E ± 2.1°	54.4°
Wh 9	-42.13901°	173.84883°	338.3°	0.81 ± 0.17	-0.93 ± 0.08	-1.71 ± 0.14	2.11 ± 0.24	76.4° E ± 1.5°	56.5°
Wh 10	-42.13983°	173.84933°	333.8°	0.45 ± 0.12	-0.71 ± 0.13	-1.57 ± 0.10	1.79 ± 0.21	86.6° E ± 2.0°	62.0°
Wh 11	-42.14064°	173.84987°	333.8°	0.29 ± 0.10	-0.71 ± 0.15	-1.47 ± 0.07	1.66 ± 0.19	87.8° W ± 2.2°	-62.5°
Wh 12	-42.14225°	173.85096°	333.8°	0.37 ± 0.17	-0.68 ± 0.20	-1.40 ± 0.10	1.60 ± 0.28	88.5° E ± 3.0°	61.0°
Wh 13	-42.14305°	173.85151°	333.8°	0.34 ± 0.13	-0.63 ± 0.34	-1.31 ± 0.10	1.49 ± 0.38	89.0° E ± 4.7°	61.4°
Wh 14	-42.14391°	173.85186°	345.0°	0.24 ± 0.18	-0.39 ± 0.22	-1.10 ± 0.14	1.19 ± 0.31	83.5° E ± 4.8°	68.3°
Wh 15	-42.14478°	173.85218°	345.0°	0.49 ± 0.18	-0.52 ± 0.21	-1.19 ± 0.14	1.39 ± 0.32	73.9° E ± 4.5°	62.8°
			**Average:**	0.44 ± 0.17	-0.83 ± 0.24	-1.16 ± 0.36	1.53 ± 0.30	81.4° E ± 12.4°	52.0° ± 12.3°
Wainui strand									
Wa 1	-42.19841°	173.87500°	177.7°	-0.03 ± 0.94	0.59 ± 0.75	-4.18 ± 0.29	4.22 ± 1.24	90.0° W ± 7.6°	-98.1°
Wa 2	-42.19931°	173.87506°	177.7°	-1.30 ± 0.73	0.01 ± 0.60	-4.23 ± 0.23	4.43 ± 0.97	72.9° W ± 4.6°	-90.8°
Wa 3	-42.20021°	173.87512°	177.7°	-1.62 ± 0.70	0.52 ± 0.41	-3.88 ± 0.45	4.24 ± 0.93	67.6° W ± 5.5°	-98.0°
			**Average:**	-0.98 ± 0.85	0.37 ± 0.32	-4.09 ± 0.19	4.29 ± 0.11	76.8° W ± 11.7°	-95.6° ± 4.19°
Edgecombe strand									
E 1	-42.18908°	173.88165°	171.9°	3.26 ± 0.69	-1.22 ± 0.47	0.52 ± 0.21	3.52 ± 0.86	9.7° W ± 8.9°	61.7°
E 2	-42.18997°	173.88183°	171.9°	2.93 ± 0.64	-1.57 ± 0.67	0.87 ± 0.22	3.44 ± 0.95	17.9° W ± 11.4°	55.1°
			**Average:**	3.09 ± 0.23	-1.40 ± 0.24	0.69 ± 0.24	3.48 ± 0.06	13.8° W ± 5.8°	58.4° ± 4.67°
Coastal strands, Far-field									
F 1	-42.18847°	173.87702°	158.7°	5.45 ± 0.47	-4.79 ± 0.47	2.44 ± 0.22	7.65 ± 0.70	36.1° W ± 2.2°	32.7°
F 2	-42.18888°	173.87724°	158.7°	6.20 ± 0.76	-4.32 ± 0.56	2.50 ± 0.32	7.96 ± 1.00	30.7° W ± 3.3°	38.0°
F 3	-42.18930°	173.87747°	158.7°	5.83 ± 0.81	-5.17 ± 0.83	2.27 ± 0.27	8.12 ± 1.19	32.6° W ± 3.0°	31.3°
F 4	-42.19014°	173.87792°	158.7°	5.33 ± 0.81	-4.54 ± 0.94	1.53 ± 0.34	7.16 ± 1.29	24.8° W ± 3.8°	30.7°
F 5	-42.19056°	173.87814°	158.7°	4.18 ± 0.77	-3.21 ± 0.75	2.59 ± 0.24	5.87 ± 1.10	43.4° W ± 5.0°	39.8°
F 6	-42.19139°	173.87859°	158.7°	3.55 ± 0.87	-4.55 ± 0.76	1.87 ± 0.78	6.07 ± 1.39	48.4° W ± 7.8°	24.3°
F 7	-42.19531°	173.87979°	149.2°	5.43 ± 4.09	-3.98 ± 0.57	1.40 ± 0.48	6.88 ± 4.16	28.1° W ± 9.9°	25.6°
F 8	-42.19570°	173.88010°	149.2°	4.59 ± 2.15	-3.52 ± 0.67	1.03 ± 0.86	5.88 ± 2.41	25.7° W ± 8.8°	23.9°
			**Average:**	5.07 ± 0.89	-4.26 ± 0.65	1.95 ± 0.58	6.95 ± 0.93	33.7° W ± 8.47°	30.8° ± 6.02°
M 33	-42.19482°	173.87950°	165.2°	3.23 ± 0.58	-3.93 ± 0.59	4.23 ± 0.15	6.62 ± 0.84	63.4° W ± 3.9°	45.6°
M 34	-42.19562°	173.88004°	149.2°	2.85 ± 0.55	-3.96 ± 0.80	3.98 ± 0.27	6.30 ± 1.00	84.0° W ± 4.7°	39.5°
			**Average:**	2.87 ± 0.65	-3.48 ± 0.62	2.83 ± 1.02	5.38 ± 1.07	57.7° W ± 16.8°	39.9° ± 7.42°
Main strand, combined									
			**Average:**	3.26 ± 0.82	-3.12 ± 0.65	6.76 ± 2.29	8.82 ± 1.92	76.2° W ± 16.0°	58.8° ± 11.8°

Table S2. Elastic forward model parameters to produce figs. S3 to S5. Elastic forward model parameters to produce fig. S3, S4, and S5 for the (M)ain strand, a lower (L)istric extension of the steeper upper Papatea main strand, the (J)ordan and (K)ekerengu faults, and the (S)ubduction interface. Depth ranges of slip are indicated next to the fault name.

label	center easting	center northing	length (km)	uniform slip (m)	strike	fault dip	rake
Papatea surface rupture (0-10km)							
1 (M 1–M 8)	734737.13	5334018.23	3.002	7.5	305.0°	84.3° E	-60.9°
2 (M 9–M 17)	736412.87	5332603.10	1.469	9.2	142.0°	80.5° W	59.3°
3 (M 18)	736977.00	5331651.02	0.767	9.0	160.0°	72.1° W	60.2°
4 (M 19–M 20)	737083.91	5331071.88	0.442	10.9	185.0°	62.0° W	70.7°
5 (M 21–M 23)	736896.09	5330341.80	1.055	10.0	196.0°	65.6° W	75.4°
6 (M 24–M 25)	736733.55	5329683.35	0.311	10.3	180.0°	74.2° W	70.1°
7 (M 26–M 27)	736845.96	5329201.63	0.679	9.9	163.0°	78.6° W	66.0°
8 (M 27–M 28)	737287.73	5327108.51	3.500	7.1	170.0°	76.7° W	47.0°
9 (M 29–M 34)	737946.70	5323955.00	2.900	5.6	168.0°	54.8° W	41.7°
Listric Papatea structure (10-20km)							
L 1	744099.26	5320647.72	3.002	7.5	125.0°	30.0° W	60.9°
L 2	748742.94	5342236.41	1.469	9.2	142.0°	30.0° W	59.3°
L 3	750217.83	5336470.29	0.767	9.0	160.0°	30.0° W	60.2°
L 4	749041.65	5330025.71	0.442	10.9	185.0°	30.0° W	70.7°
L 5	749185.15	5326817.97	1.055	10.0	196.0°	30.0° W	75.4°
L 6	751233.55	5329683.35	0.311	10.3	180.0°	30.0° W	70.1°
L 7	751481.40	5333676.13	0.679	9.9	163.0°	30.0° W	66.0°
L 8	752017.11	5329705.70	3.500	7.1	170.0°	30.0° W	47.0°
L 9	747988.64	5326089.48	2.900	5.6	168.0°	30.0° W	41.7°
Jordan and Kekerengu ruptures (0-20km)							
J	730799.32	5333087.05	6.327	6.0	223.0°	60.0° W	-165.0°
K	736998.75	5339467.22	11.427	9.0	224.0°	55.0° W	140.0°
Subduction interface (15-35km)							
S	282165.51	5308061.26	121.152	2.0	225.0°	25.0° W	125.0°
